# Influence of Physical and Technical Aspects on Change of Direction Performance of Rugby Players: An Exploratory Study

**DOI:** 10.3390/ijerph182413390

**Published:** 2021-12-20

**Authors:** Tomás T. Freitas, Pedro E. Alcaraz, Julio Calleja-González, Ademir F. S. Arruda, Aristide Guerriero, Valter P. Mercer, Lucas A. Pereira, Felipe P. Carpes, Michael R. McGuigan, Irineu Loturco

**Affiliations:** 1 UCAM Research Center for High Performance Sport, Catholic University of Murcia (UCAM), 30107 Murcia, Spain; tfreitas@ucam.edu (T.T.F.); palcaraz@ucam.edu (P.E.A.); 2 NAR—Nucleus of High Performance in Sport, São Paulo 04753-060, Brazil; valterpreis@gmail.com (V.P.M.); lucasa_pereira@outlook.com (L.A.P.); 3 Department of Human Movement Sciences, Federal University of São Paulo, Santos 11015-020, Brazil; 4 Faculty of Sport Sciences, Catholic University of Murcia (UCAM), 30107 Murcia, Spain; 5 Department of Physical Education and Sports, University of Basque Country, 01007 Vitoria-Gasteiz, Spain; julio.calleja.gonzalez@gmail.com; 6 Brazilian Rugby Confederation, São Paulo 01407-911, Brazil; ademirf.schultz@gmail.com (A.F.S.A.); guerrieroaristide1@gmail.com (A.G.); 7 Applied Neuromechanics Research Group, Federal University of Pampa, Uruguaiana 96400-100, Brazil; carpes@unipampa.edu.br; 8 School of Medical and Health Sciences, Edith Cowan University, Perth 6027, Australia; michael.mcguigan@aut.ac.nz; 9 Sports Performance Research Institute New Zealand (SPRINZ), Auckland University of Technology, Auckland 0632, New Zealand; 10 University of South Wales, Pontypridd CF037 1DL, UK

**Keywords:** muscle strength, power, team-sports, resistance training, agility, athletic performance

## Abstract

We examined the relationships between change of direction (COD) speed and deficit, and a series of speed- and power-related measurements in national team rugby union players and analyzed the influence of movement patterns on COD ability. Eleven male athletes completed the following physical assessments on different days: day 1—anthropometric measurements, and lower-body kinematic parameters (assessed with eight inertial sensors) and completion time in COD tests (pro-agility, 45° cutting maneuver (CUT), and “L” (L-Drill)); day 2—bilateral and unilateral squat and countermovement jumps, 40 m linear sprint, and bar-power output in the jump squat and half-squat exercises. Pearson’s product–moment correlations were performed to determine the relationships between COD velocities, COD deficits, and the speed–power variables. Differences between players with higher and lower COD deficits were examined using magnitude-based inferences. Results showed that (1) greater sprint momentum was associated with higher COD deficits, particularly in drills with sharper angles and multiple directional changes (L-drill and pro-agility); (2) higher unilateral jump heights were associated with greater COD deficits in the pro-agility and L-drill but not in the CUT; (3) faster athletes were less efficient at changing direction and presented greater trunk and knee flexion angles during COD maneuvers, probably as a consequence of higher inertia.

## 1. Introduction

Change of direction (COD) ability is commonly described as a multifaceted skill, underpinned by a myriad of physical and technical qualities [1,2,3,4,5]. For a better understanding of its multidimensional nature, Hewitt et al. [6] proposed dividing the COD technique into “ground-based” and “aerial-based” elements. Multiple factors such as body positioning, foot placement, stride adjustments, and joint-body segment sequencing might play a critical role in COD performance [6]. Interestingly, despite the extensive body of research on the effects of physical capacities (e.g., linear speed, strength, and muscle power) on COD speed [2,3,7,8], few studies have deeply examined the movement patterns (e.g., joint angles in different COD tasks) more associated with superior abilities to change direction.

Indeed, the vast majority of studies investigating COD technique have focused on analyzing biomechanical variables (e.g., ground reaction forces and contact times) aiming to identify how they may relate to or predict orthopedic injuries [9,10,11,12,13]. In contrast, performance-oriented investigations on COD ability are scarce in the literature. From the few studies published, aspects such as shorter ground contact times [14,15], greater ankle power output, smaller pelvis lateral tilt, and larger thorax lateral rotation [15] have been associated with better performance in “shallow” cutting maneuvers (i.e., 45° and 75° directional changes). Concerning 180° directional changes, a recent study identified that faster athletes presented greater ankle dorsiflexion and hip and knee flexion angles in the penultimate foot contact [16]. However, these studies are difficult to compare because athletes from different sports were assessed. Furthermore, it is unclear whether faster athletes (in different COD tests) displayed more effective techniques to change direction, or whether these technical factors are able to explain their superior performances. Therefore, it remains unclear which technical and biomechanical parameters (i.e., joint angles) can be used to discriminate between athletes who are more or less efficient at changing direction.

Recently, the “COD deficit” has been implemented as a complementary approach to evaluate COD efficiency in team-sport athletes [8,17,18,19]. Briefly, this measurement reports the additional time required to perform a directional change when compared to the time needed to cover the same distance in a linear sprint [19] or, alternatively, the difference in velocity between a linear sprint and a COD task of equivalent distance [8]. The COD deficit may be used as an indicator of an athletes’ efficiency to change direction since it allows evaluation of COD as a separate skill, independent of the ability to sprint over straight courses [18,19]. Previous investigations with elite soccer [18] and handball players [8] have shown that faster and more powerful athletes tend to present greater COD deficits. Nonetheless, in both cases, the authors did not analyze the technical execution of the COD maneuvers, making it impossible to determine whether certain biomechanical measures (e.g., joint angles at specific time points) are factors able to fully or at least partially explain these conflicting results.

Undoubtedly, it is of interest for practitioners to understand the movement strategies utilized by top-performers, as this could provide relevant insights to optimize training practices, and, ultimately, sport-specific performance. Furthermore, it would be useful to examine the associations between certain COD-derived outcomes (i.e., COD speed and COD deficit) and a wide range of physical performance measures such as linear sprint speed, sprint momentum, vertical jump height, and power output in different exercises. Thus, the aims of the present study were to (1) analyze the relationships between COD speed, COD deficit, and a series of speed- and power-related measurements in national rugby union players, and (2) investigate the influences of biomechanical aspects (assessed by variations in joint angles) on COD efficiency (assessed by means of COD deficit calculation).

## 2. Methods

### 2.1. Participants

A convenience sample of 11 rugby union players (age: 19.0 ± 1.9 years; body mass: 84.7 ± 9.7 kg; height: 181.7 ± 5.5 cm; sitting height: 98.8 ± 5.5 cm; leg length: 92.3 ± 5.6 cm) with heights between 175 and 185 cm from the Brazilian national team participated in the study. The criterion based on an average height of approximately 180 cm was used to avoid possible influences of anthropometric factors on movement patterns. All players were tested in the final phase of their preparation for an important international competition. The study was approved by the Federal University of São Paulo Ethics Committee and all participants were informed of the inherent risks and benefits associated with study participation, before signing informed consent forms.

### 2.2. Study Design

A cross-sectional comparative study design was used. National team rugby union players completed a series of physical tests on different days, separated by 48 h, in the following order: day 1—anthropometric measurements (height, sitting height, and leg length) and COD tests (pro-agility, 45° cutting maneuver (CUT), and “L” (L-Drill) tests); day 2—bilateral and unilateral squat and countermovement jumps (SJ and CMJ), 40 m linear sprint, and maximum bar-power output assessments in the jump squat (JS) and half-squat (HS) exercises. All players were familiarized with the testing procedures due to constant training and assessments at our sports facilities. Prior to data collection, athletes performed a standardized warm-up protocol which included general (i.e., running at a moderate pace for 10 min followed by active lower limb stretching for 3 min) and specific exercises (i.e., submaximal attempts at each test).

### 2.3. Anthropometric Measurements

Height, sitting height, and leg length were measured to the nearest 0.1 cm. Height was considered as the standing height of the athlete in the upright position, barefoot. Sitting height was measured with the players sitting on a box, erect, with their back and buttocks touching the wall, and feet on the ground. The distance from the crown of the head to the box was considered. Both heights were measured using a stadiometer. Leg length was measured with a measuring tape and consisted of the distance from the great trochanter of the femur to the ground, with athletes in the upright position, barefoot.

### 2.4. Change of Direction Ability and Change of Direction Deficit Calculation

**Figure 1:**
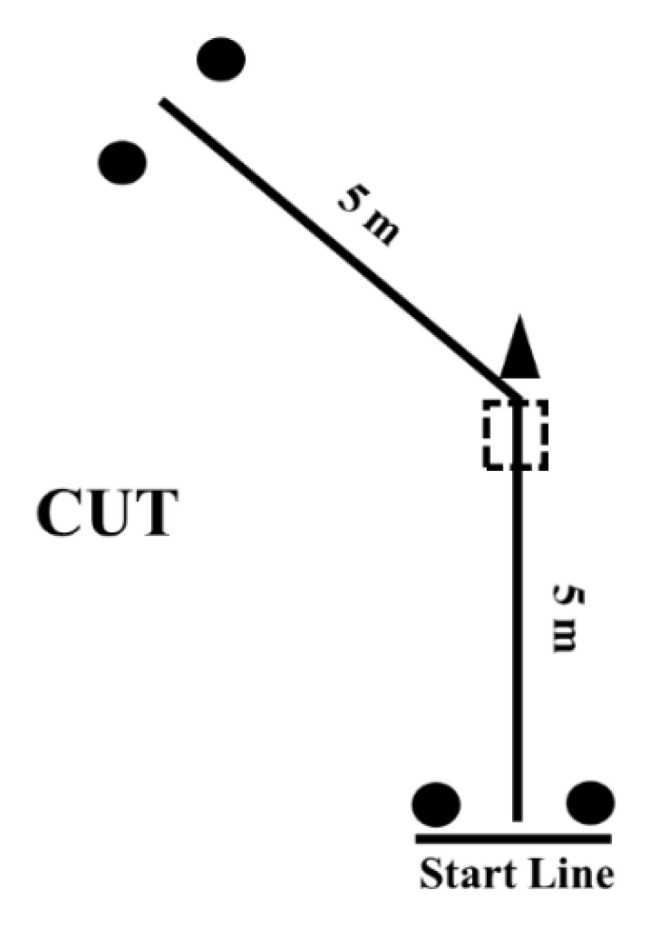
Schematic representation of the CUT test. Circles represent the position of the photocells.

Players performed three COD tasks on a synthetic indoor court with the aim of completing different maneuvers with dissimilar angles of directional change: CUT (45°), L-drill (90°), and pro-agility (180°) tests. For all tests, trials were separated by a 5 min resting interval. The CUT test consisted of a single COD action. Players started 0.3 m behind the first pair of timing gates (Smart Speed, Fusion Equipment, Brisbane, Australia) and sprinted 5 m before performing a 45° directional change and sprinting another 5 m to the finishing line (Figure 1). A cone was placed on the sprint path to “force” players to change direction. In addition, athletes were instructed to step on a 30 × 30 cm square drawn on the floor to ensure that they performed the 45° COD maneuver (avoiding a “curved sprinting” action). White tape was placed on the floor to guide the athletes in the intended direction [14]. Players performed two trials and the fastest was retained for analysis.

**Figure 2:**
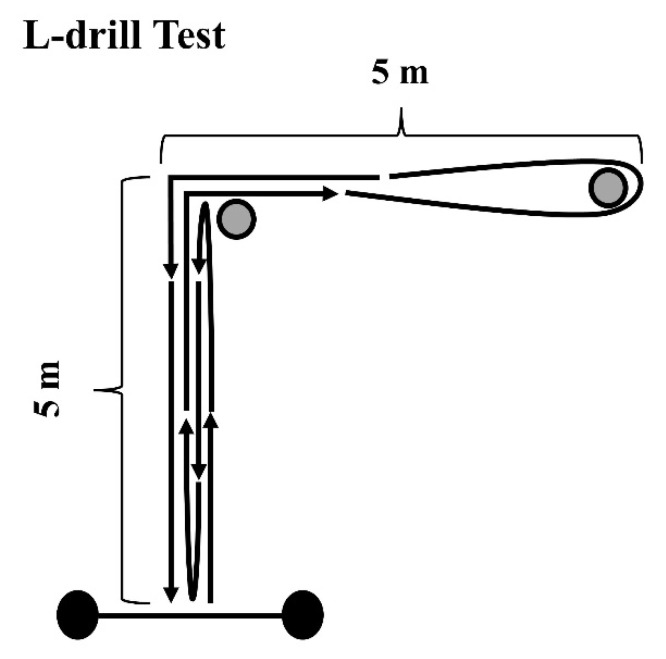
Schematic representation of the L-drill test. Black circles represent the position of the photocells and grey circles the position of the cones.

In the L-drill, participants started in a standing position, 0.3 m behind the starting line, and were required to sprint forward 5 m, cut 90° at the first cone, sprint 5 m, circle a second cone, and sprint another 5 m in the opposite direction. Then, a final 90° cut was performed before completing the 20 m test by sprinting through the photocells placed on the finishing line (Figure 2). Athletes performed two attempts to each side and the fastest was considered for analysis.

**Figure 3:**
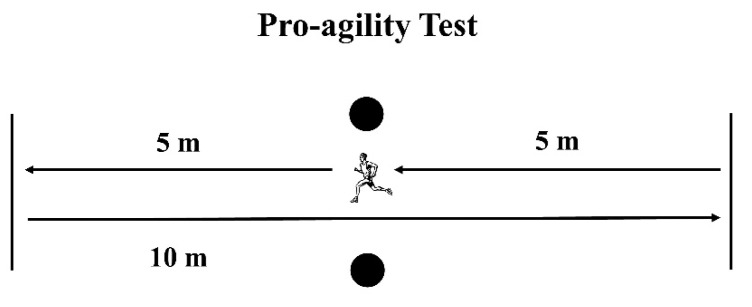
Schematic representation of the pro-agility test. Circles represent the position of the photocells.

In the pro-agility test (5-10-5), participants started in a standing position over the starting line, facing one of the photocells. At the instructor’s signal, athletes turned and sprinted 5 m, crossing the line with one foot, then turned 180° and ran 10 m towards the other line. Finally, after a second 180° COD, they sprinted 5 m through the finishing line, covering a total distance of 20 m (Figure 3). Athletes performed two attempts starting to the right side and two to the left. The fastest time of the four attempts was considered for analysis.

To evaluate COD as a separate quality (isolating linear sprint ability), an adapted COD deficit calculation was used, as described previously [8,19]. Each COD deficit was calculated based on the difference between linear sprint and COD velocities of tests of equal distance, as follows: (I) CUT: 10 m velocity—CUT velocity; (II) L-drill: 20 m velocity—L-drill velocity; (III) pro-agility: 20 m velocity—pro-agility test velocity [8,19].

### 2.5. Change of Direction Kinematics

**Figure 4:**
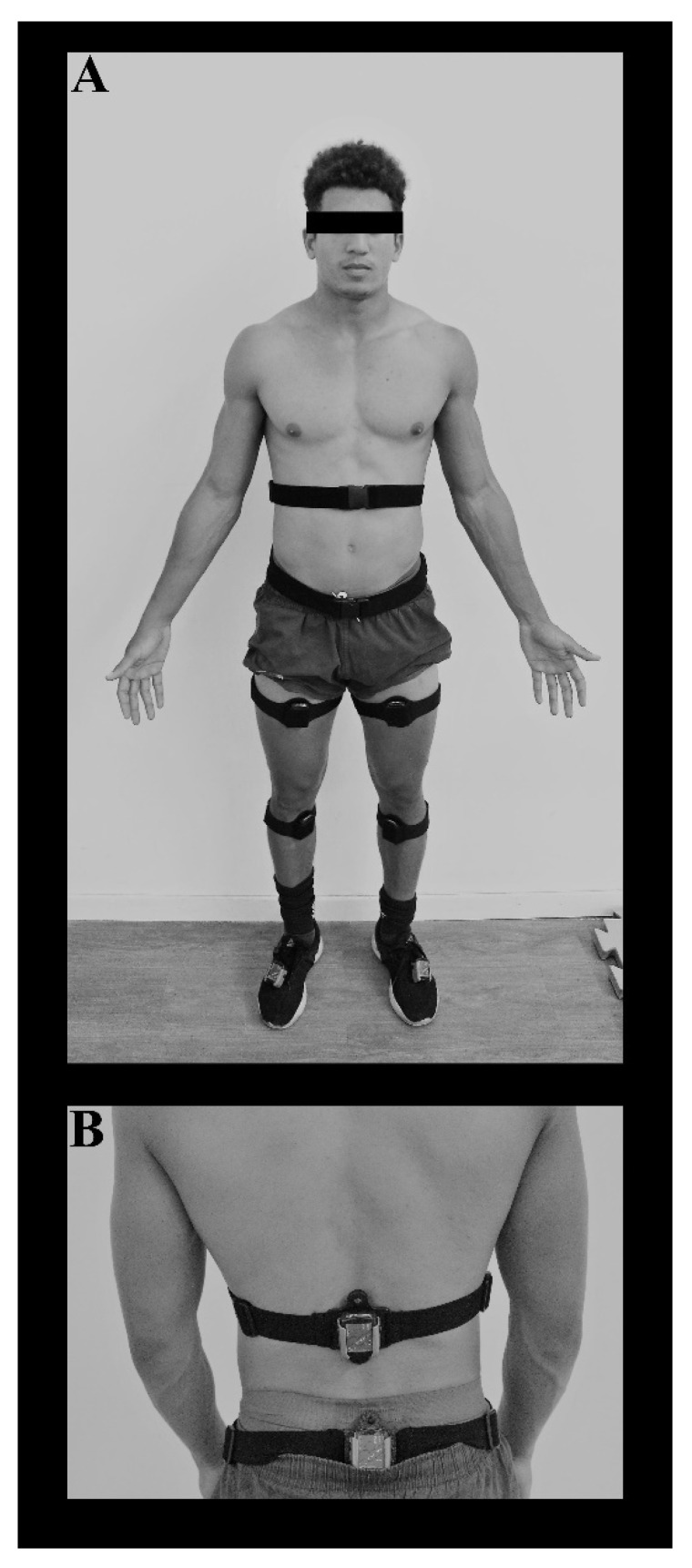
Inertial sensor placement: (**A**) front view; (**B**) back view.

Lower-body kinematic parameters were assessed in all COD tests with the Noraxon MyoMotion (Scottsdale, AZ, USA) capture and motion analysis system. A root-mean-squared error of 0.50 has been observed for the MyoMotion when compared to the Vicon Motion Capture System (Vicon, Centennial, CO, USA) with correlation coefficients of 0.99 between both systems during dynamic trials [20]. Moreover, this system has been used to assess kinematic parameters in previous research with national-team-level athletes [21]. To collect the data, eight inertial sensors were placed according to manufacturer’s guidelines on the athletes’ feet (strapped to the top of the shoe, below the ankle), shanks (frontal attachment on the tibia bone), thighs (frontal placement on the quadriceps, on the area of lowest muscle belly displacement in relation to the underlying bone), pelvis (bony area of the sacrum), and lower thorax (on the spinal cord at approximately L1/T12) (Figure 4). Prior to each COD task, the system was calibrated with the athlete in the upright position in order to determine the 0° angle of the joints studied and to allow the creation of a 3D biomechanical model, generated using Noraxon MR3.10 software. Instantaneous changes in joint angles were recorded with the software at a sampling frequency of 200 Hz. Positive values of the angle (depending on joint and axis) were considered for flexion and dorsiflexion.

For all COD tests, kinematic variables were assessed in both limbs, and legs were classified as plant leg (PL) and push-off leg (POL) [14]. The PL was considered the support leg that decelerated the body and initiated the COD maneuver and the POL was defined as the leg that initiated the acceleration of the body in the new direction [14]. Trunk, hip, and knee flexion and ankle dorsiflexion were analyzed during the contact phase of the PL and POL in each COD maneuver. In the pro-agility test, the two 180° COD actions (one for each side) were examined. The same occurred with the L-drill, in which two 90° directional changes were performed and analyzed. The initiation and completion of each COD maneuver was determined by video analysis, synchronized with the biomechanical model in MR3.10 software. Raw data were extracted to a customized spreadsheet to determine the peak angle achieved in the joint movements analyzed. In the tests in which more than one COD maneuver was performed (i.e., pro-agility and L-drill), the average of the peak angles of the PL and POL of each maneuver was considered for analysis.

### 2.6. Vertical Jumps

Bilateral and unilateral SJ and CMJ were performed on a portable force plate (AccuPower, AMTI, Graz, Austria) sampling at 400 Hz, as described previously [22]. All jumps were executed with the hands on the hips and five attempts at each jump were allowed, interspersed by 15 s intervals. Jump height, peak force (PF), and peak power (PP) were obtained from the force plate’s custom-designed software and normalized to the athlete’s BM. Leg dominance was determined based on the preferred kicking leg. The best SJ and CMJ attempts were retained for analyses.

### 2.7. Linear Sprint Velocity

The 40 m linear sprint test was conducted on an indoor running track. Five pairs of photocells (Smart Speed, Fusion Equipment, Brisbane, AUS) were positioned at the starting line and at distances of 5, 10, 20, and 40 m. Two maximal sprints, from a standing position, 0.3 m behind the starting line, were performed and a 3 min rest interval was allowed between trials. The fastest time was considered for analysis. Sprint momentum (kg·m·s^−1^) was obtained by multiplying the athlete’s body mass by the respective velocity during the linear sprint.

### 2.8. Bar-Power Outputs in Half-Squat and Jump Squat Exercises

Maximal bar-mean, mean propulsive, and peak power outputs (MP, MPP, and PP, respectively) were assessed in the HS and JS exercises on a Smith machine (Hammer Strength Equipment, Rosemont, IL, USA) using a linear encoder (T-Force, Dynamic Measurement System; Ergotech Consulting, Murcia, Spain), as previously described [23,24,25]. For the JS, athletes executed a knee flexion in a controlled manner, until the thigh was parallel to the ground, which was visually inspected by an experienced researcher. Then, they jumped as fast as possible, without losing contact with the barbell. The HS was performed in a similar manner, except that subjects were required to move the barbell as fast as possible without losing foot contact with the ground. The barbell displacement was recorded during all repetitions to verify the consistency of the eccentric action across all attempts. Both tests started at a load corresponding to 40% of the athlete’s BM and, subsequently, loads of 10% of BM were gradually added in each set, until a clear decrement (≥5%) in the bar power was observed. Furthermore, the maximal bar-mean, mean propulsive, and peak velocity (MV, MPV, and PV) achieved with the initial load in the HS and JS exercises were obtained. The HS 1-repetition maximum (1RM) was estimated based on the MPV, as reported elsewhere [26,27]. All strength-power variables were normalized to athletes’ BM.

### 2.9. Statistical Analysis

Data are presented as mean ± standard deviation (SD). The statistical analysis was performed with SPSS^®^ software package version 22.0 for Windows (SPSS, Inc., Chicago, IL, USA) and data normality was confirmed using the Shapiro–Wilk test. The Pearson product–moment coefficient of correlation was used to analyze the relationships between the COD velocities and COD deficits with sprint velocities, sprint momentum, unilateral and bilateral vertical jump variables, 1RM in the HS exercise, and bar-power outputs in the JS and HS exercises. The threshold used to qualitatively assess the correlations was based on the following criteria: <0.1, trivial; 0.1–0.3, small; 0.3–0.5, moderate; 0.5–0.7, large; 0.7–0.9, very large; >0.9 nearly perfect [28]. The significance level was set as *p* < 0.05.

Additionally, athletes were divided, using a median split analysis, into two groups according to their COD deficit values in the three COD tasks performed (e.g., higher and lower CUT COD deficit, higher and lower L-Drill COD deficit, and higher and lower pro-agility COD deficit). Due to the large inter-individual variability in the dependent variables, data were log-transformed for the analysis and then back-transformed to facilitate their presentation and interpretation. Magnitude-based inferences were used to analyze the differences between groups in the angles of the different body segments and joints assessed [29]. The magnitudes of the differences were expressed as standardized mean differences (Cohen’s d, effect size (ES)) and the smallest worthwhile change (SWC) was set by using Cohen’s principles for a small ES (i.e., 0.2) for each variable [28]. The quantitative chances of finding differences in the variables tested were assessed qualitatively as follows: <1%, almost certainly not; 1% to 5%, very unlikely; 5% to 25%, unlikely; 25% to 75%, possibly; 75% to 95%, likely; 95% to 99%, very likely; >99%, almost certainly. A meaningful difference was considered using a clinical inference, based on threshold chances of harm and benefit of 0.5% and 25% [28]. Additionally, ESs were qualitatively interpreted using the following thresholds: <0.2, trivial; ≥0.2, small; ≥0.6, moderate; ≥1.2, large; ≥2.0, very large; ≥4.0, almost perfect [28]. All tests used herein presented high levels of reliability and consistency (ICC > 0.90 and CV < 5%, for all performance measures) [28].

## 3. Results

The statistical power calculated based on the sample size (11 rugby players) for the correlations between linear sprint and COD velocities was 0.86. Table 1 shows the descriptive data of the unilateral and bilateral vertical jump heights, relative PF and PP.

Table 2 displays the descriptive data of bar-power outputs in the HS and JS exercises and the HS 1RM.

Sprint velocity and sprint momentum across the different distances tested are presented in Table 3.

Table 4 shows the COD velocity and COD deficit data for the three distinct protocols.

Table 5 shows the correlation coefficients between COD velocity and COD deficit in the three different protocols and the unilateral and bilateral vertical jumps.

Table 6 displays the correlation coefficients between COD velocity and COD deficit in the three drills and bar-power outputs in the HS and JS exercises and 1RM in the HS exercise. Large and very large significant relationships (*p* < 0.05) were found between the L-drill and pro-agility velocities and the bar-power outputs in the JS exercise.

Table 7 shows the correlation coefficients between COD velocity and COD deficit in the three distinct protocols performed and sprint velocity and sprint momentum in the different distances tested. The velocities in the CUT were largely to very largely associated with the sprint velocity in the 5, 10, 20, and 40 m tests (*p* < 0.05). Very large to nearly perfect significant correlations were found between the L-drill and pro-agility deficits and sprint velocity and sprint momentum in all distances tested (*p* < 0.05).

Finally, Table 8 shows the comparisons of joint and segment angles between lower and higher COD deficit groups for the PL and POL for the three distinct COD tasks. No meaningful differences were found in players’ standing and sitting height or leg length in any of the lower and higher COD deficit groups.

## 4. Discussion

The main findings of the present study were that (1) greater sprint momentum was associated with higher COD deficits in national team rugby union players, particularly in drills with sharper angles and multiple directional changes (i.e., L-drill and pro-agility tests); (2) higher unilateral jump heights of the dominant leg were associated with greater COD deficits in the pro-agility and L-drill but not in the CUT; and (3) athletes who were less efficient at changing direction presented greater trunk and knee flexion angles in directional change maneuvers of 45°, 90°, and 180°.

In line with previous research with different team-sports [8,18,30], higher sprint velocities were associated with greater COD deficits in the L-drill and pro-agility tests. Notably, significant relationships between all sprint velocities and COD speed (but not deficit) were found only in the less aggressive cutting maneuver (i.e., CUT), possibly because in 45° directional changes braking requirements are limited and velocity maintenance is key [4]. Accordingly, weaker relationships were identified between the COD deficit of the CUT and sprint momentum, when compared with the relationships obtained from the remaining COD tasks (Table 7). Havens et al. [31] reported different whole-body strategies when soccer players performed 45° and 90° cutting maneuvers, highlighting that changes in body momentum were indeed greater in sharper cutting angles. This is due to the fact that when performing abrupt COD, higher braking forces must be applied to reduce velocity and redirect the body into the new direction [4,31]. These findings, together with the very large correlations between sprint momentum and COD deficits observed here, suggest that sprint momentum (hence, inertia) is a mechanical variable that plays a key role in the inefficiency to change direction, especially in maneuvers with more intense decelerations. From an applied perspective, if a player is required to perform mainly 45° directional changes during competition, top speed development and velocity maintenance may be appropriate [4]. Conversely, if an athlete is demanded to regularly execute sharper COD tasks (i.e., 90° or 180°), training interventions designed to develop sprint speed (and increase inertia) should be combined with strategies focused on enhancing deceleration capacities. This “mixed training scheme” may be the most appropriate to improve COD ability and potentially attenuate the negative effects of higher levels of sprint momentum [32,33,34].

Concerning vertical jump ability, moderate to large significant relationships were found between the pro-agility and L-drill COD deficits and unilateral jump height (CMJ and SJ) of the dominant leg, and between the COD velocity in the CUT and unilateral jump performance (Table 5). Interestingly, bilateral SJ and CMJ were not related to any COD outcome (i.e., velocity or deficit), which supports previous research that also found no associations between bilateral vertical jump ability and COD deficit in different tests (i.e., 505 and zig-zag) in soccer players [18,35]. This is probably because COD maneuvers are unilateral “in nature” [36]. As expected, based on recent evidence with team-sport athletes [37,38], bar-power outputs, specifically in the JS exercise, were better associated with improved COD speed than HS 1RM measures, but no relationship was found with any COD deficit (Table 6). Considering the previous finding, important practical implications can be drawn. Firstly, coaches are advised to include unilateral jump testing in rugby players’ assessments, as this may provide better insight into potential COD inefficiencies. Secondly, the bar-power approach seems to be more effective than HS 1RM measurements to assess the functional performance of these athletes.

A unique feature of the present study was the breakdown of some critical movement strategies (i.e., joint angles) of athletes more and less efficient at changing direction, using the median split analysis for every COD task. Remarkably, in all tests, athletes with a higher COD deficit presented greater peak knee flexion angles in the POL (Table 8), which can be considered an indication of increased loading on this leg [39]. Additionally, PL peak knee flexion was greater in the CUT and L-drill, but not in the pro-agility. However, in the latter test, a 20.3% meaningfully higher trunk flexion was observed in the higher deficit group, which might have been a strategy used to compensate the lower knee flexion of the PL in the 180° turns. To some extent, these results support the findings of Dos’Santos et al. [16] but contrast with those obtained by Green et al. [14], who found no differences in PL and POL peak flexion angles between rugby players of dissimilar levels (starter vs. non-starters) during a CUT maneuver. Notably, altogether, the present findings suggest that the players with greater COD deficits used a movement pattern capable of lowering their center of mass during the COD maneuvers. Paradoxically, this strategy has been associated with better COD ability [16,40], which makes it interesting to discuss why the players who present greater COD deficits usually adopt this strategy.

The key aspect here is that, probably, this movement pattern was not “adopted” but rather “imposed” by a greater sprint momentum that may have caused, at least in part, increased loading and less than optimal peak knee and trunk flexion angles during the COD drills. From a mechanical perspective, if an athlete enters a COD with higher momentum, he or she needs to overcome higher inertia. Different studies have reported that higher approaching velocities [4,39,41] and sharper angles of directional change [4,31,39,41] require greater braking forces and decelerations. Therefore, it may be argued that the faster athletes were not able to effectively decelerate their bodies (despite lowering their center of mass during the COD maneuvers), which made them less efficient at changing direction, due to an impaired ability to cope with greater velocities [4,18] or simply counteract the associated mechanical consequences of being faster (inertia). A compelling body of evidence [4,42,43] has suggested that eccentric strength may play an important role in the capacity to decelerate and tolerate higher braking forces. However, whether faster athletes may attain an optimal body-segment position and become more efficient at changing direction (i.e., decreased COD deficit), as a result of eccentric training, remains to be determined.

Some limitations of the present study should be considered. Firstly, the cross-sectional design used did not allow confirmation of causal relationships between the assessed variables or determination of the influence of different training schemes on COD efficiency. Secondly, COD ability is a complex phenomenon in which multiple factors come together to achieve an optimal technical execution [4,15,31,40,44]. Thus, using only the peak flexion angles of the PL and POL leg to describe the movement strategy used by athletes might be an “oversimplification” of the COD technique. Aspects such as pelvis lateral tilt and larger thorax lateral rotation [15], medio-lateral step width and whole-body rotation [44], among others, have been shown to be important determinants of COD performance and additional studies should investigate whether these outcomes may differentiate rugby players with higher and lower COD deficits. Lastly, it would be interesting to identify the differences between faster and slower athletes when executing distinct COD maneuvers, when matched for COD deficit, to better understand and define what factors characterize players with different COD speeds but similar COD efficiency.

Nevertheless, to the authors’ knowledge, this is the first study to simultaneously investigate the main determinants of COD speed and deficit, and the movement strategies used by team-sport athletes more or less efficient at changing direction. These findings may have important implications in future interventions aimed at improving COD ability and reducing COD deficit.

## 5. Conclusions

Faster rugby union players appear to be less efficient at changing direction. Moreover, sprint momentum seems to be closely associated with higher COD deficits in drills with sharper angles and multiple directional changes (i.e., L-drill and pro-agility tests) while sprint velocities are more related to COD velocities (but not deficit) in less aggressive maneuvers (i.e., CUT).

### Practical Applications

Interventions aimed at increasing linear speed may differently affect COD efficiency, depending on the angle of directional change. In this regard, for players whose in-match role demands less sharp cutting maneuvers (e.g., 45°), velocity maintenance and a greater sprint momentum may be more beneficial. However, for athletes who are required to perform directional changes of more than 90°, sprint momentum might lead to increased lower-body loading and trunk and knee flexion angles during the COD maneuvers, which, in some particular situations, could hamper sport-specific performance (e.g., faster and sharper directional changes, directly associated with greater inertia). It remains unknown to what extent increasing sprint momentum is beneficial (due to the superior COD velocities achieved) or detrimental to COD performance as, despite the movement patterns adopted by faster and more powerful rugby players, these athletes still presented greater COD deficits. Therefore, practitioners are advised to quantitatively (i.e., by the COD deficit calculation) and qualitatively (i.e., using movement pattern analysis) evaluate rugby union players to better understand and monitor their technical and physical performance during different COD maneuvers. This comprehensive strategy may allow coaches and researchers to develop more effective and tailored COD training interventions.

## Figures and Tables

**Table 1:** Descriptive data (mean ± standard deviation) of the unilateral and bilateral vertical jumps.

	Height (cm)	Peak Force (N·kg^−1^)	Peak Power (W·kg^−1^)
SJ bilateral	34.8 ± 5.0	23.9 ± 1.9	54.1 ± 6.7
SJ dominant leg	16.8 ± 2.7	19.0 ± 1.3	31.2 ± 3.4
SJ non-dominant leg	16.4 ± 2.9	19.6 ± 1.2	31.6 ± 3.7
CMJ bilateral	37.6 ± 4.8	24.6 ± 2.1	52.9 ± 6.9
CMJ dominant leg	17.5 ± 3.1	18.7 ± 1.2	31.5 ± 3.7
CMJ non-dominant leg	17.6 ± 3.2	19.0 ± 1.3	32.0 ± 3.9

SJ: squat jump; CMJ: countermovement jump; peak force and peak power values were normalized by dividing them by the athletes’ body mass.

**Table 2:** Descriptive data (mean ± standard deviation) of the bar-power outputs and one repetition maximum (1RM) in the half-squat and jump squat exercises.

	MP (W·kg^−1^)	MPP (W·kg^−1^)	PP (W·kg^−1^)	1RM (kg·kg^−1^)
Half-squat	6.55 ± 0.88	7.62 ± 1.28	17.99 ± 2.40	1.68 ± 0.20
Jump squat	7.12 ± 1.37	9.92 ± 1.75	22.41 ± 3.82	-

MP: mean power; MPP: mean propulsive power; PP: peak power; values were normalized by dividing the absolute power and 1RM load by the athletes’ body mass.

**Table 3:** Descriptive data (mean ± standard deviation) of the sprint velocity and momentum in the different distances tested.

	5 m	10 m	20 m	40 m
Velocity (m·s^−1^)	4.81 ± 0.31	5.65 ± 0.31	6.58 ± 0.29	7.41 ± 0.31
Momentum (kg·m·s^−1^)	875.4 ± 71.6	1027.5 ± 74.4	1196.1 ± 67.5	1347.4 ± 71.4

**Table 4:** Descriptive data (mean ± standard deviation) of the change of direction velocity and deficit for the three distinct protocols performed.

	Cut	L-Drill	Pro-Agility
Velocity (m·s^−1^)	5.37 ± 0.27	3.35 ± 0.13	3.96 ± 0.18
Deficit (m·s^−1^)	0.28 ± 0.21	3.23 ± 0.25	2.62 ± 0.22

**Table 5:** Correlation coefficients between change of direction velocity and deficit in the three different protocols performed and unilateral and bilateral vertical jumps.

		COD Velocity			COD Deficit		
		Cut	L-Drill	Pro-Agility	Cut	L-Drill	Pro-Agility
SJ bilateral	Height	0.52	0.24	0.18	−0.03	0.46	0.52
	Peak power	0.51	0.41	0.27	−0.20	0.26	0.32
	Peak force	0.37	0.55	0.42	−0.23	0.06	0.04
SJ dominant leg	Height	0.84 *	0.49	0.51	−0.01	0.65 *	0.62 *
	Peak power	0.77 *	0.54	0.54	−0.16	0.52	0.47
	Peak force	0.54	0.57	0.53	−0.25	0.33	0.27
SJ non-dominant leg	Height	0.64 *	0.39	0.67 *	−0.17	0.44	0.19
	Peak power	0.53	0.39	0.64 *	−0.31	0.26	0.01
	Peak force	0.54	0.64 *	0.64 *	−0.50	0.07	−0.07
CMJ bilateral	Height	0.27	0.15	0.04	−0.15	0.14	0.21
	Peak power	0.32	0.27	0.13	−0.27	0.10	0.16
	Peak force	−0.05	0.35	0.17	−0.32	−0.39	−0.38
CMJ dominant leg	Height	0.86 *	0.44	0.58	0.00	0.76 *	0.66 *
	Peak power	0.75 *	0.44	0.56	−0.17	0.58	0.47
	Peak force	0.55	0.44	0.65 *	−0.60 *	0.13	−0.12
CMJ non-dominant leg	Height	0.63 *	0.21	0.50	0.11	0.60	0.41
	Peak power	0.58	0.19	0.47	−0.04	0.52	0.33
	Peak force	0.44	0.31	0.39	−0.34	0.23	0.13

COD: change of direction; SJ: squat jump; CMJ: countermovement jump; * *p* < 0.05.

**Table 6:** Correlation coefficients between change of direction velocity and deficit in the three distinct tested protocols and bar-power outputs and one repetition maximum in the half-squat and jump squat exercises.

	Half-Squat				Jump Squat		
	MP	MPP	PP	1RM	MP	MPP	PP
Cut velocity	0.36	0.28	0.46	0.39	0.39	0.50	0.46
L-drill velocity	0.50	0.55	0.54	0.53	0.61 *	0.66 *	0.77 *
Pro-agility velocity	0.26	0.41	0.50	0.41	0.54	0.64 *	0.63 *
Cut deficit	0.10	0.04	0.15	0.01	−0.07	−0.09	−0.29
L-drill deficit	0.15	0.07	0.36	0.12	0.12	0.22	0.07
Pro-agility deficit	0.25	0.07	0.32	0.12	0.06	0.12	0.01

MP: mean power; MPP: mean propulsive power; PP: peak power; 1RM: one-repetition maximum; * *p* < 0.05.

**Table 7:** Correlation coefficients between change of direction velocity and deficit for the three distinct protocols and sprint velocity and momentum over the different distances.

	Sprint Velocity				Sprint Momentum			
	5 m	10 m	20 m	40 m	5 m	10 m	20 m	40 m
Cut velocity	0.65 *	0.78 *	0.89 *	0.89 *	0.46	0.50	0.61 *	0.63 *
L-drill velocity	0.11	0.21	0.50	0.54	−0.01	0.05	0.25	0.28
Pro-agility velocity	0.25	0.39	0.65 *	0.73 *	0.09	0.17	0.35	0.42
Cut deficit	0.53	0.52	0.21	0.11	0.71 *	0.72 *	0.58	0.54
L-drill deficit	0.92 *	0.95 *	0.90 *	0.86 *	0.83 *	0.83 *	0.85 *	0.85 *
Pro-agility deficit	0.92 *	0.90 *	0.79 *	0.70 *	0.87 *	0.85 *	0.83 *	0.79 *

* *p* < 0.05.

**Table 8:** Comparison of the joint angles between lower and higher COD deficit groups for the plant and push-off legs in the three distinct change of direction protocols performed.

			Dorsiflexion	Knee Flexion	Hip Flexion	Trunk Flexion
Cut	Plant leg	Lower deficit	22.9 ± 3.2	56.8 ± 4.7	42.0 ± 8.2	29.8 ± 13.3
		Higher deficit	17.8 ± 8.3	63.1 ± 8.2	36.4 ± 13.5	28.0 ± 7.0
		Effect size	0.82 *^L^*	0.94 *^L^*	0.49	0.15
	Push-off leg	Lower deficit	27.8 ± 3.5	46.6 ± 6.6	50.5 ± 6.5	32.1 ± 8.0
		Higher deficit	27.0 ± 11.3	57.2 ± 7.9	41.2 ± 10.0	35.2 ± 12.1
		Effect size	0.09	1.42 *^VL^*	1.08 *^L^*	0.29 *^P^*
L-drill	Plant leg	Lower deficit	8.8 ± 8.7	95.5 ± 14.6	54.2 ± 17.9	33.6 ± 5.5
		Higher deficit	10.2 ± 17.1	102.0 ± 16.2	62.3 ± 17.7	33.0 ± 8.5
		Effect size	0.10	0.41 *^P^*	0.44 *^P^*	0.08
	Push-off leg	Lower deficit	21.3 ± 10.1	54.6 ± 10.3	41.8 ± 19.5	37.8 ± 3.7
		Higher deficit	24.2 ± 5.9	57.6 ± 3.2	39.0 ± 12.2	32.4 ± 9.7
		Effect size	0.32 *^P^*	0.34 *^P^*	0.16	0.73
Pro-agility	Plant leg	Lower deficit	8.8 ± 9.9	113.0 ± 6.0	86.5 ± 8.0	40.5 ± 8.7
		Higher deficit	19.3 ± 11.6	110.1 ± 7.6	81.6 ± 8.8	42.1 ± 7.0
		Effect size	0.95 *^L^*	0.51	0.56	0.19
	Plant leg	Lower deficit	26.3 ± 11.3	55.4 ± 10.5	39.3 ± 12.8	45.9 ± 11.8
		Higher deficit	22.8 ± 15.8	70.0 ± 9.1	41.1 ± 15.2	55.2 ± 10.9
		Effect size	0.25	1.42 *^VL^*	0.13	0.79 *^L^*

*^P^*: possibly different; *^L^*: likely different; *^VL^*: very likely different.

## Data Availability

All relevant data are within the manuscript.

## References

[1] Condello G., Kernozek T.W., Tessitore A., Foster C. (2016). Biomechanical analysis of a change-of-direction task in collegiate soccer players. Int. J. Sports Physiol. Perform..

[2] Condello G., Minganti C., Lupo C., Benvenuti C., Pacini D., Tessitore A. (2013). Evaluation of change-of-direction movements in young rugby players. Int. J. Sports Physiol. Perform..

[3] Delaney J.A., Scott T.J., Ballard D.A., Duthie G.M., Hickmans J.A., Lockie R.G., Dascombe B.J. (2015). Contributing factors to change-of-direction ability in professional rugby league players. J. Strength Cond. Res..

[4] Dos’ Santos T., Thomas C., Comfort P., Jones P.A. (2018). The effect of angle and velocity on change of direction biomechanics: An angle-velocity trade-off. Sports Med..

[5] Sheppard J.M., Young W.B. (2006). Agility literature review: Classifications, training and testing. J. Sports Sci..

[6] Hewit J.K., Cronin J.B., Hume P.A. (2013). Kinematic factors affecting fast and slow straight and change-of-direction acceleration times. J. Strength Cond. Res..

[7] Chaouachi A., Brughelli M., Chamari K., Levin G.T., Ben Abdelkrim N., Laurencelle L., Castagna C. (2009). Lower limb maximal dynamic strength and agility determinants in elite basketball players. J. Strength Cond. Res..

[8] Pereira L.A., Nimphius S., Kobal R., Kitamura K., Turisco L.A.L., Orsi R.C., Abad C.C.C., Loturco I. (2018). Relationship between change of direction, speed, and power in male and female national olympic team handball athletes. J. Strength Cond. Res..

[9] Cortes N., Onate J., Van Lunen B. (2011). Pivot task increases knee frontal plane loading compared with sidestep and drop-jump. J. Sports Sci..

[10] Dempsey A.R., Lloyd D.G., Elliott B.C., Steele J.R., Munro B.J. (2009). Changing sidestep cutting technique reduces knee valgus loading. Am. J. Sports Med..

[11] Dempsey A.R., Lloyd D.G., Elliott B.C., Steele J.R., Munro B.J., Russo K.A. (2007). The effect of technique change on knee loads during sidestep cutting. Med. Sci. Sports Exerc..

[12] Jones P.A., Herrington L.C., Graham-Smith P. (2015). Technique determinants of knee joint loads during cutting in female soccer players. Hum. Mov. Sci..

[13] Kristianslund E., Faul O., Bahr R., Myklebust G., Krosshaug T. (2014). Sidestep cutting technique and knee abduction loading: Implications for acl prevention exercises. Br. J. Sports Med..

[14] Green B.S., Blake C., Caulfield B.M. (2011). A comparison of cutting technique performance in rugby union players. J. Strength Cond. Res..

[15] Marshall B.M., Franklyn-Miller A.D., King E.A., Moran K.A., Strike S.C., Falvey E.C. (2014). Biomechanical factors associated with time to complete a change of direction cutting maneuver. J. Strength Cond. Res..

[16] Dos’Santos T., McBurnie A., Thomas C., Comfort P., Jones P.A. (2020). Biomechanical determinants of the modified and traditional 505 change of direction speed test. J. Strength Cond. Res..

[17] Dos’ Santos T., Thomas C., Jones P.A., Comfort P. (2019). Assessing asymmetries in change of direction speed performance; application of change of direction deficit. J. Strength Cond. Res..

[18] Loturco I., Nimphius S., Kobal R., Bottino A., Zanetti V., Pereira L.A., Jeffreys I. (2018). Change-of direction deficit in elite young soccer players. Ger. J. Exerc. Sport Res..

[19] Nimphius S., Callaghan S.J., Spiteri T., Lockie R.G. (2016). Change of direction deficit: A more isolated measure of change of direction performance than total 505 time. J. Strength Cond. Res..

[20] Balasubramanian S., Abbas J. (2013). Comparison of Angle Measurements between Vicon and Myomotion Systems.

[21] Bankosz Z., Winiarski S., Malagoli Lanzoni I. (2020). Gender differences in kinematic parameters of topspin forehand and backhand in table tennis. Int. J. Environ. Res. Public Health.

[22] Bishop C., Read P., Lake J., Loturco I., Turner A. (2021). A novel approach for athlete profiling: The unilateral dynamic strength index. J. Strength Cond. Res..

[23] Loturco I., Nakamura F.Y., Tricoli V., Kobal R., Abad C.C., Kitamura K., Ugrinowitsch C., Gil S., Pereira L.A., Gonzales-Badillo J.J. (2015). Determining the optimum power load in jump squats using the mean propulsive velocity. PLoS ONE.

[24] Loturco I., Pereira L.A., Abad C.C., Tabares F., Moraes J.E., Kobal R., Kitamura K., Nakamura F.Y. (2017). Bar velocities capable of optimising the muscle power in strength-power exercises. J. Sports Sci..

[25] Sanchez-Medina L., Perez C.E., Gonzalez-Badillo J.J. (2010). Importance of the propulsive phase in strength assessment. Int. J. Sports Med..

[26] Gonzalez-Badillo J.J., Sanchez-Medina L. (2010). Movement velocity as a measure of loading intensity in resistance training. Int. J. Sports Med..

[27] Loturco I., Pereira L.A., Cal Abad C.C., Gil S., Kitamura K., Kobal R., Nakamura F.Y. (2016). Using bar velocity to predict the maximum dynamic strength in the half-squat exercise. Int. J. Sports Physiol. Perform..

[28] Hopkins W.G., Marshall S.W., Batterham A.M., Hanin J. (2009). Progressive statistics for studies in sports medicine and exercise science. Med. Sci. Sports Exerc..

[29] Batterham A.M., Hopkins W.G. (2006). Making meaningful inferences about magnitudes. Int. J. Sports Physiol. Perform..

[30] Freitas T.T., Alcaraz P.E., Bishop C., Calleja-González J., Arruda A.F.S., Guerriero A., Reis V.P., Pereira L.A., Loturco I. (2018). Change of direction deficit in national team rugby union players: Is there an influence of playing position?. Sports.

[31] Havens K.L., Sigward S.M. (2015). Whole body mechanics differ among running and cutting maneuvers in skilled athletes. Gait Posture.

[32] Dos’Santos T., Thomas C., Jones P.A., Comfort P. (2017). Mechanical determinants of faster change of direction speed performance in male athletes. J. Strength Cond. Res..

[33] Fernandes R., Bishop C., Turner A.N., Chavda S., Maloney S.J. (2020). Train the engine or the brakes? Influence of momentum on the change of direction deficit. Int. J. Sports Physiol. Perform..

[34] Harper D.J., Kiely J. (2018). Damaging nature of decelerations: Do we adequately prepare players?. BMJ Open Sport Exerc. Med..

[35] Lockie R.G., Stage A.A., Stokes J.J., Orjalo A.J., Davis D.L., Giuliano D.V., Moreno M.R., Risso F.G., Lazar A., Birmingham-Babauta S.A. (2016). Relationships and predictive capabilities of jump assessments to soccer-specific field test performance in division i collegiate players. Sports.

[36] Thomas C., Dos’Santos T., Comfort P., Jones P.A. (2018). Relationships between unilateral muscle strength qualities and change of direction in adolescent team-sport athletes. Sports.

[37] Loturco I., Pereira L.A., Moraes J.E., Kitamura K., Cal Abad C.C., Kobal R., Nakamura F.Y. (2017). Jump-squat and half-squat exercises: Selective influences on speed-power performance of elite rugby sevens players. PLoS ONE.

[38] Loturco I., Suchomel T., James L.P., Bishop C., Abad C.C.C., Pereira L.A., McGuigan M.R. (2018). Selective influences of maximum dynamic strength and bar-power output on team sports performance: A comprehensive study of four different disciplines. Front. Physiol..

[39] Nedergaard N.J., Kersting U., Lake M. (2014). Using accelerometry to quantify deceleration during a high-intensity soccer turning manoeuvre. J. Sports Sci..

[40] Nimphius S., Callaghan S.J., Bezodis N.E., Lockie R.G. (2018). Change of direction and agility tests: Challenging our current measures of performance. Strength Cond. J..

[41] Jones P.A., Thomas C., Dos’Santos T., McMahon J.J., Graham-Smith P. (2017). The role of eccentric strength in 180 degrees turns in female soccer players. Sports.

[42] Chaabene H., Prieske O., Negra Y., Granacher U. (2018). Change of direction speed: Toward a strength training approach with accentuated eccentric muscle actions. Sports Med..

[43] de Hoyo M., Sanudo B., Carrasco L., Mateo-Cortes J., Dominguez-Cobo S., Fernandes O., Del Ojo J.J., Gonzalo-Skok O. (2016). Effects of 10-week eccentric overload training on kinetic parameters during change of direction in football players. J. Sports Sci..

[44] David S., Mundt M., Komnik I., Potthast W. (2018). Understanding cutting maneuvers—The mechanical consequence of preparatory strategies and foot strike pattern. Hum. Mov. Sci..

